# An evaluation of the clinical skills and experience within an orthopaedic Integrated Clinical Assessment and Treatment Service

**DOI:** 10.3399/bjgpopen17X101217

**Published:** 2017-12-13

**Authors:** Simon Feist-Wilson, Neil Heron

**Affiliations:** 1 Medical Student, Department of General Practice and Primary Care and Centre for Public Health Research, Queen’s University, Belfast, Northern Ireland; 2 NIHR Clinical PhD Fellow in GP and Sport and Exercise Medicine, Department of General Practice and Primary Care and Centre for Public Health Research, Queen's University, Belfast, Northern Ireland

**Keywords:** general practice, integrated clinical assessment service, musculoskeletal, orthopaedics, primary care, integrated care

## Abstract

**Background:**

General practice in the UK is ‘in crisis’. With 20% of GP workload relating to musculoskeletal (MSK) problems, an orthopaedic Integrated Clinical Assessment and Treatment Service (ICATS) could help support assessment of these patients in primary care, alleviating pressure on GPs. However, practitioners in ICATS must be trained appropriately to ensure its effectiveness.

**Aim:**

This evaluation aimed to identify the training levels of doctors in one Northern Ireland orthopaedic ICATS system, what their future training needs are, and suggestions for how this service could be improved to better support general practice.

**Design & setting:**

A questionnaire study in an orthopaedic ICATS, Northern Ireland.

**Method:**

All seven doctors working within the Southern Trust orthopaedic ICATS were asked to complete a questionnaire detailing their training and experience in MSK medicine. Their views on how the service could be improved were elicited.

**Results:**

Six of seven questionnaires were returned. All responders were Members of the Royal College of General Practitioners (MRCGP), while five of six held a Diploma in Sports and Exercise Medicine (Dip SEM). Half of responders suggested that MSK ultrasound could be beneficial within ICATS. However, it was viewed that extensive training would be required before paediatric MSK patients could be included.

**Conclusion:**

High levels of training and experience were reported by responders, suggesting ICATS provides a high-level MSK service. Furthermore, it was noted that inclusion of MSK ultrasound and paediatric patients into this service could be beneficial but not without undertaking further training. With appropriate funding and support the ICATS service has the potential to expand the clinical services it offers to general practice, helping to reduce work pressures in primary care at this time of crisis for UK general practice.

## How this fits in

General practice in the UK is currently experiencing significant workload and staffing pressures, and more support needs to be provided. Orthopaedic ICATS is one such service which can better support both primary and secondary care by reviewing MSK patients in the community. This study characterised the clinical experience and educational qualifications of one orthopaedic ICATS in Northern Ireland to identify potential additional support which this service could provide to GPs and to secondary care.

## Introduction

The increasing workload on the general practice service within the UK is a well-known issue for the NHS.^1,2^ The number and duration of consultations for GPs has risen over recent years,^3^ driving the service to potential breaking point, with the British Medical Association Northern Ireland (NI) reporting that general practice is ‘in crisis’.^1^ With this greater demand, waiting times for appointments inevitably increase: a common source of health service complaints.^4^ Easing the pressure on GP services is therefore of paramount importance if the NHS is to withstand the demand being placed on its shoulders. One possible solution is to redistribute certain patient groups into other services within the healthcare system. Given that 20% of GP workload is related to MSK problems,^5–7^ a significant burden on the GP service could be removed if this patient group were treated within a more specialised service, outside of the traditional general practice setting. The orthopaedic ICATS system provides both assessment and treatment of MSK conditions in the community setting, being traditionally housed outside hospital premises, and helps build capacity into primary care.

ICATS aims to provide a service whereby patients suffering from orthopaedic conditions can obtain access to specialist assessment and treatment on referral from their GP. Management solutions for orthopaedic ailments within this service include joint injections, exercise prescription, pain management, podiatry, physiotherapy, and further referral to orthopaedic surgery. The additional benefits which ICATS offers over the traditional primary-to-secondary care referral pathway include: reduced patient waiting times for orthopaedic conditions (the waiting time for patients to be reviewed in the Southern Trust, NI, ICATS is currently approximately 24 weeks compared to >1 year for routine orthopaedic secondary care referrals); shortening the duration of GP MSK appointments by offering treatment within ICATS; helping to better utilise limited resources such as different imaging modalities; and improving the quality of onward referrals to secondary care. Patient follow-up can also be maintained within this service, further relieving pressure on the GP service.

However, in order to maximise the potential benefits of ICATS, the service needs to be sustainable. The efficient and effective operation of this service is reliant on numerous factors, but particularly the level of workforce training within ICATS. Healthcare workers, and GPs in particular, do not feel they have the skills or knowledge required to treat patients with MSK problems.^8,9^ Thus, ensuring that those working in ICATS are trained to a suitable standard is fundamental to its success. This evaulation therefore aimed to identify the training levels of doctors in one Northern Irish orthopaedic ICATS system, what their future training needs are, and how this service could be improved to better support general practice in managing the current pressures within UK primary care.

## Method

In order to assess the level of training in the current ICATS setup, a questionnaire was devised and distributed to all doctors working within ICATS (further information available from the authors on request). This study was conducted within the Southern Trust, NI, orthopaedic ICATS and there are currently seven GPs with a specialist interest (GPwSIs) in orthopaedics or MSK medicine working in the department. The questionnaire aimed to assess the current qualifications and training held by these practitioners and their views on what training was necessary to carry out their role. This included the use of radiology equipment and joint injections. In addition, participants were asked about their confidence regarding the use of MSK ultrasound and provision of paediatric MSK care, and their opinions on the practicality of its potential incorporation into ICATS. Furthermore, subjects were also asked to identify areas where they would like more training.

The questionnaire was distributed to all seven doctors working within the service by mail. Prior to distribution, the questionnaire was piloted with two doctors from this service to assess its appropriateness. Participants were encouraged to complete the questionnaire individually, with all questionnaire responses being anonymised and all data treated confidentially.

## Results

### Responder data

Of the seven questionnaires distributed, six (86%) were returned over a 6-week period. All responders were male. The key information from responders can be seen in **Table 1**. The average age of responders was 43 years, the mean time spent practising as a GP was 14 years, and the mean time working for ICATS was 8 years. All six responders were MRCGP, with five out of the six also holding a Dip SEM, and three possessing Fellowship of the Faculty of Sport and Exercise Medicine (FFSEM) in either Ireland or the UK. Every responder stated that prospective candidates for ICATS should at least hold a diploma in sports and exercise medicine (SEM), orthopaedic medicine, or MSK medicine in order to work effectively within ICATS.

**Table 1:** Summary of responder characteristics

**Characteristics**	**Responders, *n***
**Sex**	
Male	6
Female	0
**Age, years**	
31–35	1
36–40	1
41–45	3
46–50	0
51–55	1
**Qualifications**	
MRCGP	6
FFSEM	3
MFSEM	2
Dip SEM	5
Masters in a relevant area	1
Orthopaedic/MSK Diploma	3
**Years as a GP**	
<5	1
5–10	1
11–15	2
16–20	1
≥21	1
**Years in ICATS**	
≤5	2
6–10	4

Dip SEM = Diploma in Sport and Exercise Medicine. FFSEM = Fellowship of the Faculty of Sport and Exercise Medicine. ICATS = Integrated Clinical Assessment and Treatment Services. MFSEM = Membership of the Faculty of Sport and Exercise Medicine. MRCGP = Membership of the Royal College of General Practitioners. MSK = musculoskeletal. SEM = Sport and Exercise Medicine.

### Experience in MSK-related areas

Five of the six responders reported prior experience working in the MSK field before starting their roles in orthopaedic ICATS. Experience working for sports teams, including rugby and Gaelic Athletic Association teams, was commonly reported, as was previous work in sports medicine clinics. Two subjects described experience working in orthopaedic settings prior to their role in ICATS.

### Ultrasound in ICATS

Participants responded favourably to the notion of using ultrasound within ICATS, with all those surveyed expressing an interest in extending its role within the service. There were, however, differing responses regarding the desired extent of ultrasound use.

Five practitioners agreed that the use of ultrasound would be beneficial for guided injections, but two additionally felt it could be extended to aid diagnosis. One response stated:

*'Eventually USS [ultrasound scan] can be used in the clinic to offer a one-stop-shop of diagnosis and intervention.'* (Male, age 44 years)

However another response suggested it should not be used for diagnosis. A further opinion was that staff within ICATS:

*'... don’t need to replicate the role of the radiology department.'* (Male, age 54 years)

It was also stated that:

*'... if used sensibly, can add [ultrasound] to inject a patient to aid management.'* (Male, age 54 years)

Four of the doctors surveyed described at least some prior experience in ultrasound, having attended at least one course in MSK ultrasound training. However, one doctor who had >2 years' experience in MSK ultrasound, having attended different training courses, still felt that further training was required before ultrasound could be fully incorporated into ICATS. In addition to further training, the responder felt that supervised MSK ultrasound with radiology consultants would be beneficial. Moreover, five responders reported some experience with radiology training, such as interpretation of X-rays and MRIs. For three of these five, this was through A&E training, while the other two reported that their experience in this aspect of MSK medicine was through diplomas they had obtained.

### Joint injections in ICATS

All six doctors described having undertaken training in joint injections at some point prior to their work in ICATS. Half reported that this was through multiple courses, with three others reporting that this training had been conducted in supervised sports medicine and orthopaedic clinics.

### Paediatric MSK in ICATS

Three participants had at least some experience in treating paediatric patients with MSK problems, but their level of experience varied from only seeing these patients within their normal GP workload to seeing patients within paediatric clinical attachments as part of their GP training. The remaining three reported no experience with paediatric patients and thus little confidence in treating this group. Those with experience still suggested that further training would be required before paediatric patients with MSK issues could be seen in ICATS. One of those surveyed, one GP suggested that the training would have to be:

*'... extensive to ensure that subtle problems are not missed.'* (Male, age 54 years)

### Views on further training

In addition to the views regarding training for ultrasound and paediatric MSK, a range of responses were obtained concerning the participants' opinions on what ongoing training and topics they would like to see covered at a local, regional, or national level within ICATS. The need for further training with the use of ultrasound-guided joint injections was echoed in this section by three out of the six practitioners. One of these three also suggested that training relating to tendinopathy and the evidence for its treatment should be covered, a view that was shared by another of the responders, who also viewed training in an alternative therapy, prolotherapy, as an important issue that should be covered in training for ICATS. Training courses in MRI and X-ray interpretation were suggested by one responder, who had previously undertaken an X-ray course for A&E training. There was also a suggestion by another responder that continued updates through regional ICATS meetings could be very useful for the service. This opinion was similar to that of the following responder, who suggested:

*'A rolling education programme covering key MSK topics, particularly when research indicates a change in practice, is required.'* (Male, age 43 years)

## Discussion

### Summary

This survey of doctors working within orthopaedic ICATS demonstrates the high-level qualifications which they possess, both in terms of GP qualifications (MRCGP) and specific MSK or SEM qualifications, with 50% of those surveyed holding FFSEM, which is equivalent to consultant status in SEM. The ICATS doctors had significant experience in MSK medicine, 8 years on average, and most had experience with MSK ultrasound, wishing to develop further expertise in this area. Potential areas for further development in orthopaedic ICATS include further MSK ultrasound work, both to guide injections and to potentially aid diagnosis; and potentially extending the patient groups covered by ICATS to include paediatric patients with MSK conditions. Such development of the service would need to be supported with the provision of extra resources, for example appropriate training resources and secondary care consultant input.

### Strengths and limitations

The response rate for the questionnaire is high but the study was only conducted in one ICAT service, within one trust. The next stage of this project will be to sample more trusts within the UK. It would also be interesting to sample GPs’ views when referring into orthopaedic ICATS, and how they feel the service could be further developed to better support their needs and those of their patients. Furthermore, the orthopaedic ICATS team is multidisciplinary, including physiotherapy and podiatry colleagues who are extremely well trained. It would therefore be of interest to conduct this audit within the full orthopaedic ICATS team, demonstrating the extensive skills and experience which they bring to their patients.

### Comparison with existing literature

MSK conditions make up a significant workload within general practice, with one in seven GP consultations reported to be for MSK conditions.^6^ The current ethos within the UK healthcare system is for a ‘shift left’ in patient management and within NI there is the *Transforming Your Care* policy,^10^ with more and more medical conditions being managed within the community. Yet general practice within the UK and in NI is in ‘crisis’,^1,2^ with significant workload pressures on those who work within the community and in the primary care team. The onus is therefore on those working within general practice and the community to develop innovative models of care, breaking down the traditional barriers between primary and secondary care, allowing more effective management of the workload within primary care by developing integrative models of care. One way to do this is to invest in orthopaedic ICATS which accept MSK referrals from primary care and manages these patients within community settings. Indeed all patients who are referred from primary care to orthopaedic ICATS in the Southern Trust, NI, currently wait ≤24 weeks to be seen by a specialist, while those with secondary care routine orthopaedic appointments generally wait >1 year (Belfast Trust Commissioner for orthopaedic conditions and the Clinical Lead for Orthopaedic ICATS, Southern Trust, personal communication, 2017). If orthopaedic ICATS could be extended to offer more appointments, then this would offer further support to primary care within NI. Moreover, ICATS clinics are costed at £900 per clinic, while secondary care orthopaedic clinics are costed at £1600 per clinic, which makes this approach feasible and sustainable (Belfast Trust Commissioner for orthopaedic conditions and the Clinical Lead for Orthopaedic ICATS, Southern Trust, personal communication, 2017).

### Implications for practice

Another option to further extend the use of orthopaedic ICATS within the community would be to consider offering additional services. At present, ultrasound examinations are not provided within ICATS. However, it can be seen from these survey results that, given the correct training, MSK ultrasound could be incorporated into this service in order to provide more accurate diagnosis and treatment for patients, further reducing secondary care referrals. Furthermore, allowing paediatric patients to use this service could further attenuate the demand on GPs and help alleviate the workload pressures within primary and secondary care. However, it has been reported that practitioner confidence and experience in paediatric MSK conditions needs to be improved,^11^ and therefore appropriate training would need to be put in place to ensure that transitioning these patients into ICATS returns favourable outcomes. Secondary care support for developing and expanding orthopaedic ICATS would be welcomed, in terms of integrating both MSK ultrasound and paediatric patients with MSK conditions into the service. Moreover, GPs often report low confidence levels in managing MSK conditions.^7^ One of the extended roles of orthopaedic ICATS must therefore be to educate primary care colleagues on these common conditions and help ensure the best possible primary care management of MSK conditions. Supporting evidence for the role that Southern Trust ICATS plays in educating its GPs within the trust is that orthopaedic referrals in the region are lower, at 5.7 per 1000 patients, compared to the Northern Ireland average of 10.3 per 1000 patients. Indeed, Southern Trust ICATS’ role in GP education has included formulating protocols for managing MSK conditions in primary care, joint injection education days, and updates related to management of spinal issues. This could be further developed within the trust, for example, through face-to-face teaching on examination skills and a regular seminar series on common MSK conditions, including pain management.

In conclusion, this study presents the first review of the medical qualifications and experience of doctors working within the orthopaedic ICATS department in the Southern Trust, NI, and future developmental needs of the service. The findings confirm the high level of physician training of those working within the system, with 50% of those responding holding consultant-level qualifications. Future training needs identified include MSK ultrasound training and paediatric MSK, and secondary care support to deliver these services would be welcomed. Investment in orthopaedic ICATS would help support Northern Irish general practice at a time when it is 'in crisis’ and help deliver better services to patients.
